# ^1^H NMR Urinary Metabolomics Profiling of Newborns with Congenital Human Cytomegalovirus Infection: Insights into Metabolic Alterations

**DOI:** 10.1021/acs.jproteome.5c00017

**Published:** 2025-03-25

**Authors:** Alessia Spadavecchia, Marta Zoccarato, Gaia Tedone, Matteo Biolatti, Valentina Dell’Oste, Agata Leone, Alessandro Cossard, Mattia Sozzi, Ilia Bresesti, Enrico Bertino, Roberto Gobetto, Alessandra Coscia, Angelo Gallo

**Affiliations:** † Neonatal Unit, Department of Public Health and Pediatric Sciences, University of Turin, Turin 10126, Italy; ‡ Department of Chemistry, University of Turin, Turin 10125, Italy; § Department of Public Health and Pediatric Sciences, University of Turin, Turin 10126, Italy; ∥ Department of Applied Science and Technology, Polytechnic of Turin, Turin 10129, Italy; ⊥ Division of Neonatology, “Filippo Del Ponte” Hospital, University of Insubria, Varese 21100, Italy

**Keywords:** cytomegalovirus, HCMV, congenital, infection, metabolomic, NMR spectroscopy

## Abstract

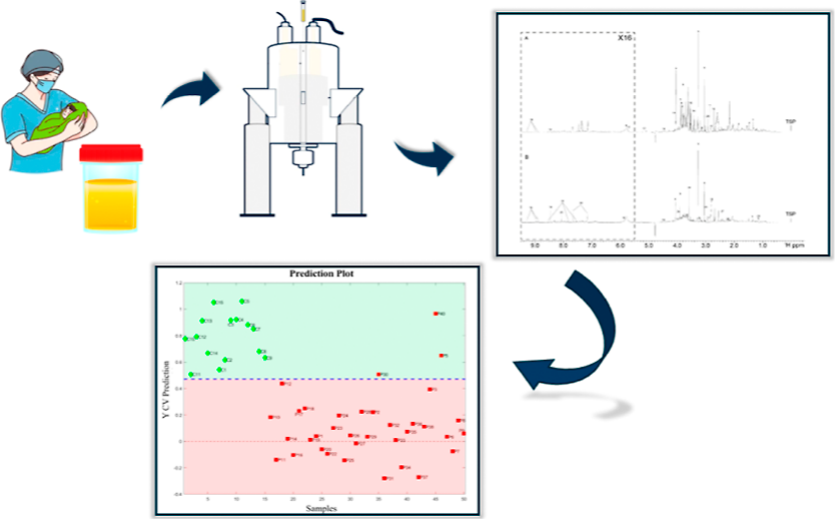

Human cytomegalovirus (HCMV) is the leading cause of congenital infections resulting in severe morbidity and mortality among newborns worldwide. Currently, the most significant prognostic factor of congenital cytomegalovirus (cCMV) infection is the time of maternal infection, with a more severe clinical phenotype if the mother’s first outbreak occurs during the first trimester of pregnancy. Nonetheless, the pathogenesis of cCMV infection has still to be completely characterized. In particular, little is known about the metabolic response triggered by HCMV in congenitally infected newborns. As such, urinary metabolic profiling by ^1^H nuclear magnetic resonance (NMR) might represent a promising tool to be exploited in the context of cCMV. This study aims to investigate the impact of HCMV infection on the urine metabolome in a population of congenitally infected newborns and uninfected controls by ^1^H NMR spectroscopy combined with multivariate statistical analysis. The ^1^H NMR spectra of patients (*n* = 35) and controls (*n* = 15) allowed the identification of an overall amount of 55 metabolites. Principal Component Analysis (PCA) and clustering correctly assigned 49 out of 50 newborns into the infected and control groups. Partial Least-Squares-Discriminant Analysis (PLS-DA) revealed that newborns with cCMV resulted in having increased betaine, citrate, 3-hydroxybutyrate, 4-hydroxybutyrate, acetoacetate, formate, glycolate, lactate, succinate, and threonine levels in the urine. On the other hand, healthy controls showed increased 4-aminohippurate, creatine, creatinine, fumarate, mannitol, taurine, and dimethylamine levels. These results showed a clear difference in metabolomic fingerprint between newborns with cCMV infection and healthy controls. Thus, metabolomics can be considered a new, promising diagnostic and prognostic tool in the clinical management of cCMV patients.

## Introduction

Human cytomegalovirus (HCMV) is a double-stranded DNA herpesvirus characterized by wide seroprevalence and lifelong latency in the infected population. The most severe clinical manifestations have been reported in immunocompromised patients and congenitally infected newborns. In developed countries, congenital HCMV infection (cCMV) is the most common type of congenital infection, leading to significant morbidity and mortality among newborns.^1,2^ The infection can either be asymptomatic or symptomatic at birth. Newborns with symptomatic cCMV may experience hearing loss, microcephaly, hepatosplenomegaly, petechiae, visual impairment, and cerebral involvement, causing developmental and motor delays.^2^ The severity of symptoms at birth is strongly associated with the timing of maternal infection during pregnancy, with a most severe clinical outcome if the infection occurs during the first trimester of gestation.^2^ Nonetheless, even symptomatic newborns (50%) and those asymptomatic (13%) can develop long-term audiological and neurodevelopmental sequelae later in infancy.^3^ The diagnosis of cCMV can be assessed during pregnancy or after birth, by identifying viral DNA in newborns’ biological materials (urine, blood, and saliva) by polymerase chain reaction (PCR). A pressing clinical issue is the early identification of asymptomatic newborns, especially those at risk of long-term neurodevelopmental complications. Due to the lack of effective HCMV vaccines and some drawbacks of pharmacological treatments,^4^ there is an urgent and unfulfilled clinical requirement to tackle HCMV infection in these particularly vulnerable patients. This scenario highlights the need for advancements in identifying and managing cCMV.

Recent in vitro studies have revealed that HCMV induces comprehensive metabolic reprogramming in host cells to enhance its replication and release. Among the most significantly affected pathways are glycolysis, the tricarboxylic acid (TCA) cycle, and pyrimidine synthesis.^5−7^ As such, interpreting how HCMV can modify the metabolic pathways for viral replication represents an important area to be exploited for the development of new diagnostic and prognostic tools, with metabolomics potentially offering a valuable contribution.

In recent years, metabolomics has made significant progress in translational diagnostics research, enabling the systematic investigation of the full range of low molecular weight metabolites found in biofluids or tissue samples.^8,9^ However, despite these advancements, the clinical implementation of metabolomics is yet to be fully realized. Urine stands out as an ideal sample for metabolomics investigations in pediatric and neonatal conditions, primarily due to its rich concentration of metabolites and noninvasive collection methods. Preclinical studies have already utilized metabolomics to explore various perinatal and neonatal conditions, including HCMV infection.^10−13^ Nonetheless, global metabolomic studies on the urine of newborns with cCMV are strongly envisaged to enhance our understanding of the biological aspects of HCMV infection and assess the feasibility and utility of implementing metabolomics approaches in a clinical hospital setting.

This observational study aims to examine the effect of HCMV congenital infection on the urine metabolome by comparing global biochemical profiles and clinical features of HCMV-infected newborns vs uninfected as controls. Specifically, urine samples were collected from a previously characterized cohort of pediatric patients with confirmed HCMV congenital infection.^14−16^ In this study, proton nuclear magnetic resonance (^1^H NMR) spectroscopy combined with multivariate statistical analysis was used to further explore and refine the functional characterization of congenital HCMV infection. The primary goal was to define a patient profile that could assist in predicting infection outcomes.

## Experimental Section

### Ethics Statement

This study was approved by the Research Ethics Committee of the University Hospital of Turin “A.O.U. Città della Salute e della Scienza di Torino – A.O. Ordine Mauriziano – A.S.L. TO1” (no. CS2/122). Informed consent was obtained from the parents of all study participants before collecting demographic and clinical data, along with biological samples. The work was carried out in accordance with the Declaration of Helsinki.

### Study Subjects

Thirty-six patients with diagnosed cCMV infection were recruited from 2015 to 2024 at the Neonatal Unit of the University of Turin. Infection diagnosis was based on RT-PCR HCMV DNA detection in patients’ urine and blood samples. In parallel, 17 healthy age-, gender-, and race-matched newborns without HCMV infection were also included in the study as normal controls. Most urine samples were collected during the initial medical examination within the first month after birth.

### Urine Sample Collection and Preparation

Urine samples (50 out of 53 patients recruited) were collected using urine collection bags and stored at −80 °C until analysis. Three samples were not analyzed since the amount was insufficient for NMR spectroscopy analysis. Before ^1^H NMR analysis, samples were thawed at 4 °C and then centrifuged at 13,000 rpm for 10 min at 4 °C. The supernatant (540 μL) was transferred into a new tube and mixed with 60 μL of phosphate buffer solution (1.5 M KH_2_PO_4_, 1% sodium 3-trimethylsilyl-propionate-2,2,3,3-*d*_4_ (TSP), pH 7.4 in D_2_O). The mixture was then centrifuged at 13,000 rpm, 4 °C, for 10 min, to ensure good homogenization. Each sample (600 μL) was placed into 5 mm NMR tubes for the acquisition of ^1^H NMR spectra and analysis.^12^

### ^1^H NMR Data Acquisition and Processing

The acquisition of ^1^H NMR spectra was conducted at 300 K (27 °C) using a JEOL (JNM-ECZ600R/M1) spectrometer operating at 600 MHz. A 1D NOESY pulse sequence was used with water suppression. For each urine spectrum, a total of 64 scans were collected with a TD of 65536 points over a spectral width of 20.025 ppm using a relaxation delay of 4 s, an acquisition time of 4.36 s, and a mixing time of 10 ms, as previously reported.^17^ All spectra were phased, and the baseline was corrected using JEOL Delta v6.0. The chemical shift scale was referenced by assigning a value of δ 0.00 ppm to the internal standard TSP signal. All the spectra were imported and processed under MATLAB environment (R2022a version, MathWorks, Natick, MA, USA).^18^ To increase the spectra comparability, the icoshift^19^ tool was applied to align the most important and characteristic signals located in specific manually selected ppm intervals. The interval selection was manually performed to better identify those ppm regions where the signals were clearly visible and potentially assignable to known metabolites. To remove uninformative and noisy areas, the spectra width was corrected by including only signals between −0.1 and 10.5 ppm. To avoid interferences during the data analysis steps, the residual water signal (4.64–5.15 ppm) and the TSP signal (−0.04–0.04 ppm) were removed from the spectra. The spectral points were then normalized to the total sum of the intensities. A downsampling approach was also applied to reduce the number of variables by selecting one out of every ten points, reducing from 26205 to 2621 variables.

### Data Analysis

The assignment of the metabolites was performed based on literature data, the Human Metabolome database (http://www.hmdb.ca),^20^ and Chenomx Profiler 9.0 software (Chenomx Inc., Edmonton, Canada). Additionally, only for metabolite identification, 2D ^1^H NMR J-RES spectra were acquired under the same experimental conditions but a total of 4 scans, an acquisition time of 0.26 s, and a relaxation delay of 2 s. Furthermore, a 2D TOCSY spectrum was included for a urine control sample to confirm, identify, and assign metabolites. A total of 512 scans were collected using an acquisition time of 0.36, and a relaxation delay of 1.5 s.

### Multivariate Statistical Analysis

^1^H NMR spectral datasets were first mean-centered, and then an exploratory Principal Component Analysis (PCA)^21^ was performed using the PLS_toolbox (version 8.9.2, eigenvector Research Inc., Manson, WA, USA) software package. Following PCA, the spectral data were subjected to Partial Least Squares-Discriminant Analysis (PLS-DA) to maximize the covariance between the variables and a target response corresponding to a “class” to which the samples belong. To evaluate which variables (i.e., the ^1^H NMR signals) mostly contribute to the discrimination ability of the PLS-DA model,^22^ an approach that combines Variable Important in Projection (VIP) scores and Selectivity Ratio (SR) was inspected.^23^ Additionally, all the PLS-DA models created were validated using only the Cross-Validation (CV) approach.^24^ The decision of not splitting the original dataset into calibration and test sets was based on the limited number of available samples. Furthermore, no replicates were included in the statistical analysis, making the cross-validation splitting more robust.

## Results and Discussion

### Characteristics of Study Subjects

A total of 53 participants were recruited, i.e., 36 newborns with cCMV infection and 17 healthy controls. Uninfected controls were included in the study during the first admission after birth: inclusion criteria comprehended having a normal perinatal clinical course and physiological pregnancy. Controls were not enrolled in the study if they had an acute illness, exposure to antibiotics or steroids, or any underlying comorbidity. The urine samples from 1 cCMV-infected infant and two healthy controls were excluded due to the low amount of urine. This resulted in a study cohort of 35 newborns with cCMV infection and 15 healthy controls.

For patients with cCMV, diagnosis was established within the first 21 days of life as part of the standard of care. Following diagnosis, all patients underwent evaluations for neurobehavioral development, growth parameters, cerebral ultrasound (cUS), vision, hearing, and the need for antiviral or supportive therapy. Asymptomatic patients received clinical and neurobehavioral follow-up for one year, while symptomatic patients were followed for two years, with hearing assessments continuing for six years. Whenever possible, urine samples were collected within the first 3 weeks of life (median age at sampling: 57.26 ± 50.50 days). During the fetal period, cCMV infection was undetected during pregnancy in 16 cases and diagnosed through maternal serology in 17 cases. In one case, it was suspected and confirmed after ultrasound abnormalities were observed. Primary maternal infection was identified in 23 patients, while maternal nonprimary infection occurred in 5 cases; 1 patient was diagnosed with postnatal cCMV infection. The timing of cCMV infection was distributed as follows: 12 cases during the first trimester, seven during the second, and six during the third. Antiviral therapy with Valacyclovir was administered during pregnancy in 4 cases. The median gestational age at birth was 38.18 ± 2.89 weeks. Preterm births occurred in 4 cases. Delivery was vaginal in 29 patients, while seven were delivered via cesarean section. Resuscitation at birth was required in 2 cases. Regarding newborn characteristics, birth weight was below the 3^rd^ percentile for gestational age in 6 cases and between the 3^rd^ and 10^th^ percentiles in 3 cases. Head circumference was below the 10^th^ percentile for gestational age in 11 patients. Clinical symptoms at birth, including petechiae, hepatomegaly, splenomegaly, chorioretinitis, hypotonia, or convulsions, were observed in 6 patients. Instrumental examinations revealed pathological hearing in 13 patients, abnormal findings on cerebral ultrasound (cUS) in 20 cases, and pathological cerebral MRI in 22 cases. Laboratory analyses showed abnormal platelet counts in 4 patients, pathological absolute neutrophil counts in 3 patients, and impaired hepatic function in 1 case. Antiviral therapy with Valganciclovir was administered for six months in 16 cases. The baseline clinical and pathological characteristics of enrolled patients are summarized in Table S1.

For healthy controls, urine samples were collected within the first 3 days of life during birth admission (median sampling age: 1.118 ± 0.582 days). All controls underwent a complete clinical examination at birth as part of standard care. During pregnancy and the fetal period, none of the mothers contracted HCMV infection, and all pregnancies progressed without complications. The median gestational age at birth was 39.758 ± 1.273 weeks, with all patients born at term. Delivery was vaginal in 12 cases, while three required cesarean sections; none of the patients required resuscitation at birth. All patients had birthweights and head circumferences above the 10^th^ percentile for gestational age. The perinatal clinical course was uneventful for all patients. The baseline clinical characteristics of enrolled controls are summarized in Table S2.

### Metabolic Profile and Pattern Recognition of Urine Samples Using ^1^H NMR Spectroscopy

**Figure 1:**
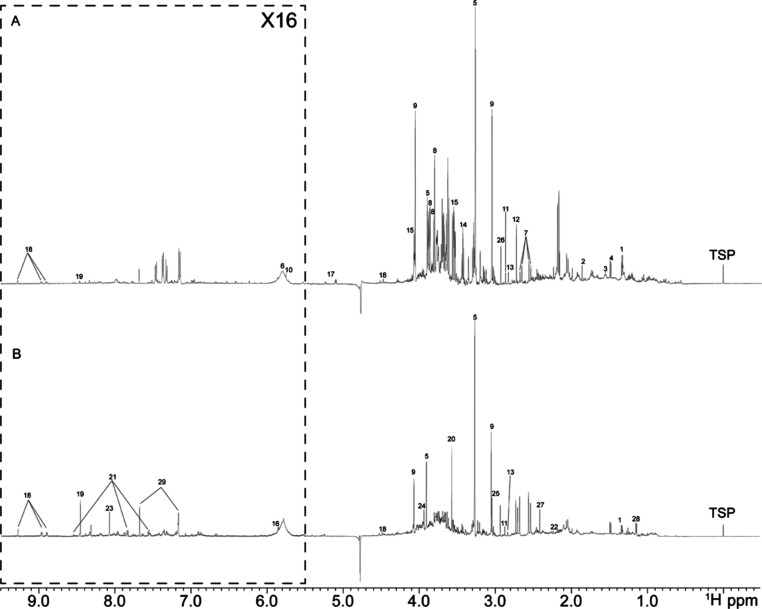
Representative urine 1D ^1^H NMR spectra (600 MHz) from (A) controls and (B) patients. Selected metabolites annotations: 1 lactate; 2 acetate; 3 adipate; 4 alanine; 5 betaine; 6 urea; 7 citrate; 8 mannitol; 9 creatinine; 10 *cis*-aconitate; 11 trimethylamine; 12 dimethylamine; 13 methylguanidine; 14 taurine; 15 myo-inositol; 16 xanthosine; 17 glucose; 18 *N*-methylnicotinamide; 19 formate; 20 glycine; 21 hippurate; 22 acetone; 23 oxypurinol; 24 glycolate; 25 creatin-phosphate; 26 *N*,*N*-dimethylglycine; 27 succinate; 28 propylene glycol; 29 histidine.

High-quality ^1^H NMR spectra were obtained from the participants’ urine samples. Representative 1D ^1^H NMR urine spectra from a control (A) and a patient (B) are reported in Figure 1.

A total of 55 endogenous and exogenous metabolites were identified (Table S3), encompassing a wide array of compounds such as organic acids, aminoacids, lipids, a lot of tricarboxylic acid (TCA) cycle metabolites, glycolysis cycle metabolites, and citric acid cycle metabolites.^7,10^ The last three classes of compounds are of particular interest since they confirmed the influence exerted by HCMV on these metabolic pathways.^7^ Highly overlapping spectral profiles have been obtained, without significant differences in the metabolites detected between the group of controls and the group of patients. The signals that can be recorded fall within a spectral window between 0 ppm, a signal assigned to the peak of the internal standard (TSP), and 9.5 ppm, typical chemical shifts in the spectral range of aromatic and amide groups. The 2–4 ppm chemical shift range contains the highest concentration of identified metabolites, including amino acid-derived compounds, alcohols, and aliphatic carbons. Among the metabolites identified, the peaks of creatinine (3.02–4.06 ppm) and betaine (3.27–3.90 ppm), both singlets, stand out in the spectrum for their high intensity. The creatinine peaks appear higher in most of the spectra acquired for controls, while peaks belonging to betaine are higher in the patients’ spectra. Likewise, the singlet of glycine (3.57 ppm), the triplets characteristic of taurine (3.25–3.42 ppm), the doublet of doublets of citrate (2.55–2.70 ppm), or the large singlet of urea (5.80 ppm) can be easily identified in the controls.

**Figure 2:**
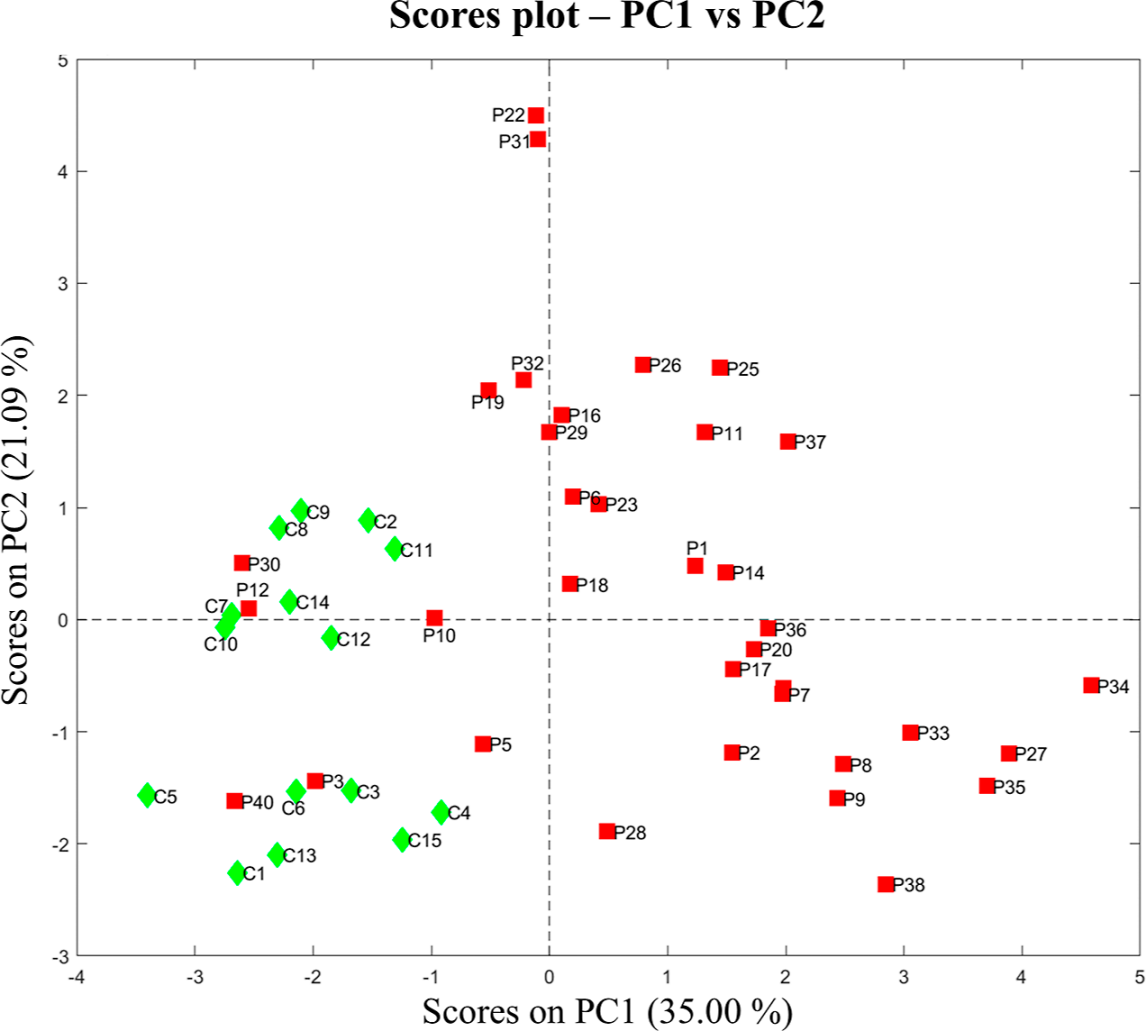
PCA scores plot (PC1 vs PC2) of ^1^H NMR spectra obtained from cCMV infected patients (red squares, “P”), or healthy controls (green rhombs, “C”).

PCA was initially applied to the dataset (all the spectra) obtained after the variable down-sampling to highlight possible metabolic differences among the urine samples from cCMV patients and uninfected controls and to identify potential outliers. This technique helped us decompose the overall data set into one described by a new set of variables, called Principal Components (PC), representing maximum variance sources. The exploration of this new “Principal Components space” provided information related to the sample distribution (“Scores plot”) and the variable effect (“Loadings plot”). The number of Principal Components was set to 2, describing a total cumulative variance of 56.09%. From the scores plot obtained considering component 1 (PC1) (35.00%) vs component 2 (PC2) (21.09%) two main clusters can be observed (Figure 2).

Notably, the two clusters can be associated with the two monitored classes, i.e., cCMV infected patients (shown in red squares and labeled with “P”), or uninfected healthy controls (shown in green rhombs and labeled with “C”). Accordingly, the scores plot, that allows us to find possible clusters, trends, or outliers among the samples, and loadings plot, which must be interpreted with the scores plot, show how the initial variables relate to the distribution of the samples. The results for PC1 (Figure S1) indicate that it is primarily responsible for the cluster’s separation. All samples labeled as “C” (controls) tend to have low PC1 score values, whereas samples labeled as “P” (infected patients) exhibit higher PC1 scores. In addition, the loadings plot revealed that some spectral signals could be associated with the “P” cluster, while others mainly belong to the spectra of samples labeled as “C”.

Since PCA revealed a clear separation between the spectra of the infected samples and the healthy participants, a classification analysis was performed with PLS-DA, aiming to build a model able to distinguish the presence of cCMV infection (“P” class) in urine samples compared to healthy controls (“C” class).

**Figure 3:**
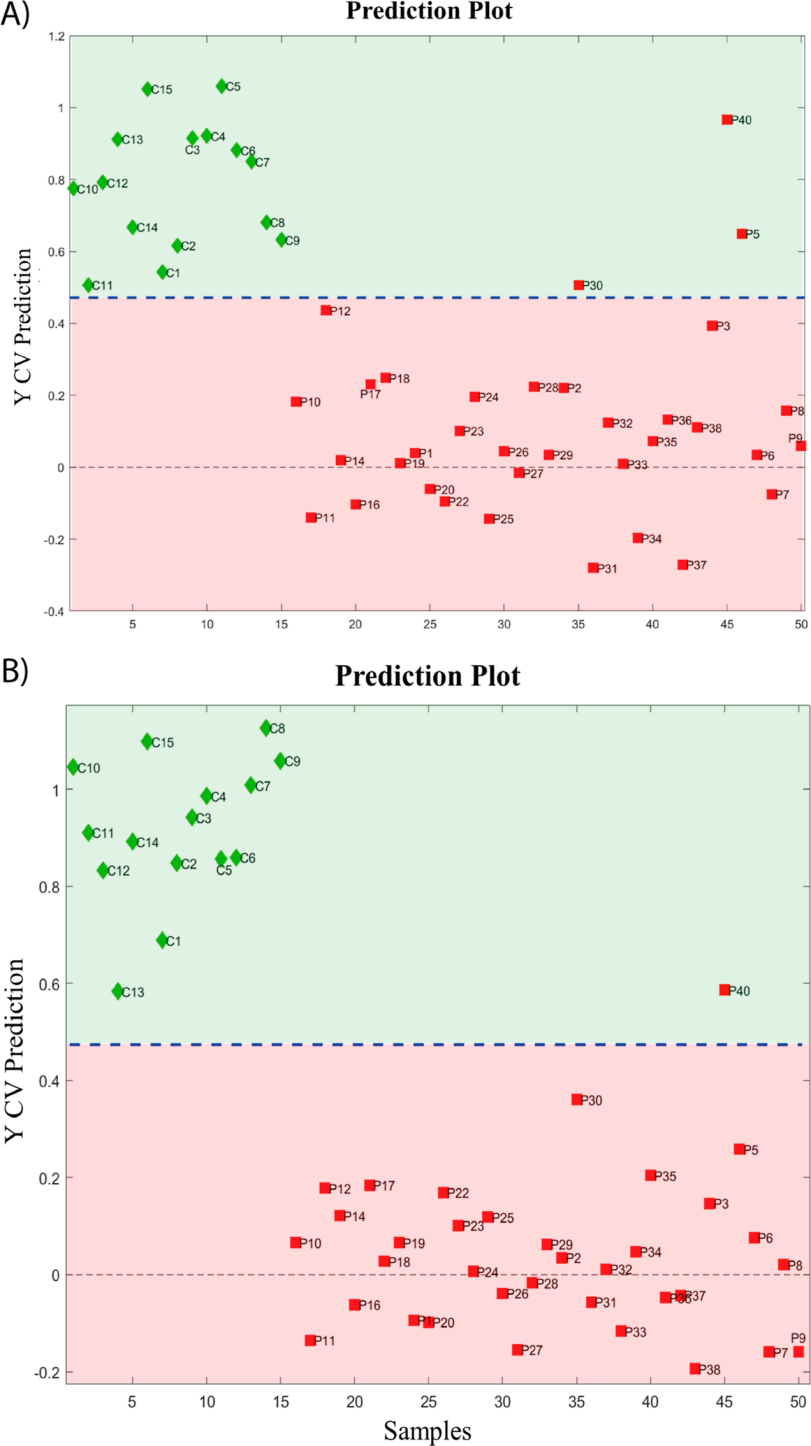
(A) Prediction plot obtained from the first PLS-DA model. The samples are colored according to the two modeled classes (red squares, “P” or green rhombs, “C”). (B) Prediction plot obtained from the second PLS-DA model built using only those variables selected with the VIP scores and SR combined approach. The samples are colored according to the two modeled classes (red squares, “P” or green rhombs, “C”).

This approach resulted in a new space definition described by a new set of variables called “Latent Variables” (LV), which are chosen based on their ability to discriminate between classes. The main outputs of a PLS-DA model are the confusion matrix, which allows evaluation of the performance and the classification ability of the model by showing the predicted class of the different samples; the prediction plot, where the samples are distributed according to the response predicted by the model; and the scores and loadings plots, which provide information related to samples and variables. All the PLS-DA models were validated using cross-validation with “venetian blinds” as a selection scheme to create the reduced subsets. In the first PLS-DA model, 2 latent variables were selected, describing a cumulative Y-covariance of 76.12% and cumulative explained variance of 47.56%. The overall classification error in cross-validation was 0.043, derived from three misclassified “P” samples wrongly assigned to the “C” class, as visible also from the confusion matrix reported in Table 1. The model performance measures reveal an overall accuracy for the two classes of 94%, with a sensitivity of 1.0 and a specificity of 0.91, defining a good discrimination ability of the noninfected samples. The prediction plot (Figure 3A) indicates that the three misclassified samples can be identified in P5, P30, and P40. The Receiver Operating Characteristic (ROC) curve obtained from this PLS-DA model is reported in the (Figure S2), and the Area Under the curve (AUROC) is 0.966.

**Table 1:** Confusion Matrix Obtained from a PLS-DA Model Performed Considering all the Samples and Working with Two Latent Variables

Real/Predicted	Control	Patient
Control	15	3
Patient	0	32

The nature of the samples might explain the misclassification obtained for these three patients. Indeed, P5 is a subject born preterm, a condition often associated with metabolic deficits and severe immune system immaturity. This scenario hinders the proper functioning of metabolic reactions responsible for the degradation and the formation of products and intermediates, leading to metabolic deficiencies. The peculiar metabolites that we report here for this patient are myo-inositol, caprylate, suberate, and sebacate. The unusually high peaks of myo-inositol could be attributed to the extended duration of breastfeeding required for premature birth. The caprylate, sebacate, and suberate can be associated with cholestasis and immaturity of the hepatic function. Combining this information, it is not surprising that P5 is misclassified. Furthermore, this patient has very strong signals of ibuprofen, which is typically associated with hospitalization.

The misclassification of P30 may be explained by the timing of urine collection, which occurred at 9 months of age. By this point, the patient’s growth likely differed significantly from the general cohort, potentially due to factors such as diet and environmental factors. Accordingly, Frick et al.^11^ reported no differences in urinary metabolites between cases and controls in babies older than 28 days of life. Indeed, it is well established that metabolite levels can be influenced by factors such as age, weight, height, sex, and exercise.^25^

The misclassification of P40, like the P30, may be attributed to the sample being collected at 9 months of age, corresponding to a significantly later time than other patients. This is reflected in P40’s creatinine peaks, which show the highest intensity. In infants, creatinine levels naturally rise as renal function matures during the first months of life, making the timing of sampling a key factor.^26^ Clinically, P40 exhibited severe neurological involvement, including polymicrogyria and ventriculomegaly, identified through imaging, which resulted in profoundly impaired neurodevelopment.

In addition, to assess any possible correlation between clinical parameters and infection within the patient cohort, we performed additional PCA, including factors such as type and trimester of infection, any antiviral therapies administered during pregnancy, fetal abnormalities (e.g., IUGR), mode of delivery and gestational age, sex, ethnicity, and presence of severe symptoms (e.g., deafness, pathological brain ultrasound). The PCA revealed good clustering overall, but only the ethnicity and fetal abnormalities parameters highlighted the separation of only one patient from the rest of the group.

In detail, upon examining clustering by ethnicity, only P37 stood apart from the rest of the patients, since he/she belongs to the Asian population. However, if we consider the clinical parameter related to fetal anomalies, only P12 is separated from the group. Similarly, this distinction can be attributed to P12 being affected by IUGR, and his metabolic profile differs from others in that this type of abnormality is characterized by a very specific metabolic pattern.

One more possible justification for the separation of P37 from the rest of the patients is given by exogenous factors such as diet and lifestyle rather than a direct correlation between infection development and ethnicity. In both cases, we should keep in mind that the number of patients included in this case study is limited, as routine screening for HCMV is still relatively uncommon.

To improve the investigation and after tentatively assigning the ^1^H NMR spectra and identifying a series of metabolites, a variable selection approach was also applied to the previously described PLS-DA model to identify the metabolites that allow distinguishing the two explored classes. This process led to the extraction of a subset of 436 variables based on an approach (implemented in the PLS_toolbox) that combines VIP scores and Selectivity Ratio methods (Figures S2 and S3). VIP scores indicate the importance of each variable included in the model, with higher VIP scores indicating greater importance. On the other hand, the Selectivity Ratio assesses variable importance by quantifying the proportion of its variance explained by the predictive components relative to the total variance. The outputs evaluation allows us to perform a variable selection approach, aiming to improve the model performances by reducing the number of variables included in the model and selecting only the most useful for classification purposes. A second PLS-DA model was then built using this new reduced dataset. Three latent variables were selected, describing a cumulative Y-covariance of 92.82% and a cumulative explained variance of 82.80%. Thanks to the variable selection approach applied, this model revealed an overall classification error in cross-validation of 0.014. According to the confusion matrixshown in Table 2, only one sample was misclassified, leading to an overall accuracy of 98.0%, a sensitivity of 1.0, and a specificity of 0.97. By exploring the prediction plot (Figure 3B), the only misclassified sample is P40. The ROC curve obtained from this PLS-DA model is reported in the (Figure S4), and the AUROC is 0.998.

**Table 2:** Confusion Matrix Obtained from a PLS-DA Model Performed Considering Only a Subset of Selected Variables and Working with Three Latent Variables

Real/Predicted	Control	Patient
Control	15	1
Patient	0	34

The cross-validation approach used overcame the problem of the low number of samples by performing an iterative process consisting of splitting the original datasets into reduced subsets with a lower number of samples, such as ours. A series of models were then built using these independent subsets (calibration subsets), and the models were then tested using the samples excluded from the calibration subsets. This iterative approach allowed us to assess the robustness and reliability of a PLS-DA model by providing a more accurate estimate of model performances. By exploring the selected variables, 18 metabolites were identified as fundamental to distinguishing between the two classes studied. Table 3 provides a list of these metabolites, along with a specification of which class contains higher concentrations of each metabolite.

**Table 3:** List of Metabolites and Their Chemical Shifts Selected Using VIP Scores and SR Combined Variable Selection Approach^a^

metabolites	ppm (multiplicity)	“class”
3-hydroxyisobutyrate	1.06 (d), 2.48 (m), 3.53 (m), 3.69 (m)	Patients (“P”)
4-aminohippurate	3.90 (d), 6.90 (d), 7.80 (d), 8.20 (s)	Controls (“C”)
4-hydroxybutyrate	1.74 (m), 2.21 (t), 3.57 (t)	Patients (“P”)
acetoacetate	2.29 (s), 3.43 (s)	Patients (“P”)
betaine	3.27 (s), 3.90 (s)	Patients (“P”)
citrate	2.55 (dd), 2.70 (dd)	Patients (“P”)
creatine	3.02 (s), 3.91 (s)	Controls (“C”)
creatinine	3.02 (s), 4.06 (s)	Controls (“C”)
creatine phosphate	3.04 (s), 3.94 (s)	Controls (“C”)
dimethylamine	2.73 (s)	Controls (“C”)
formate	8.46 (s)	Patients (“P”)
fumarate	6.53 (s)	Controls (“C”)
glycolate	3.94 (s)	Patients (“P”)
lactate	1.32 (d), 4.10 (q)	Patients (“P”)
mannitol	3.71 (m), 3.82 (m), 3.90 (dd)	Controls (“C”)
succinate	2.41 (s)	Patients (“P”)
taurine	3.25 (t), 3.42 (t)	Controls (“C”)
threonine	1.32 (d), 3.58 (d), 4.25 (m)	Patients (“P”)

a Interestingly, newborns with cCMV resulted in having increased 3-hydroxyisobutyrate, 4-hydroxybutyrate, acetoacetate, betaine, citrate, formate, glycolate, lactate, succinate, and threonine. On the other hand, healthy controls showed increased 4-aminohippurate, creatine, creatinine, creatine phosphate, dimethylamine, fumarate, mannitol, and taurine.

These differences highlight the impact of HCMV on metabolic pathways, particularly those related to energy production and amino acid metabolism.^6,27^ Metabolites such as citrate and lactate in infected newborns suggest enhanced glycolysis and tricarboxylic acid (TCA) cycle activity, aligning with previous in vitro studies indicating that HCMV infection induces metabolic reprogramming to provide the virus with energy and precursor molecules for its replication, thus affecting normal cellular metabolic balance and establishing a specific metabolic signature for the virus.^7,28^ Moreover, the increased lactate observed in cCMV newborns is consistent with recent research showing that HCMV infection induces metabolic reprogramming in glioblastoma cells. This reprogramming causes the surrounding tumor microenvironment to become permissive to tumor progression, akin to the reverse-Warburg effect.^29^

The identification of increased betaine and threonine levels in cCMV patients is particularly interesting. Betaine is known for its role in methyl group donation, influencing DNA methylation and gene expression, while threonine is essential for protein synthesis and immune function. The upregulation of these metabolites may reflect a host response to viral infection, potentially linked to immune activation and epigenetic modifications that could influence the severity of the disease. Even though the literature contains few reports describing the ^1^H NMR urine spectra of HCMV congenitally infected newborns in a hospital setting, our finding that betaine is a discriminant metabolite in infected patients is in agreement with the findings reported by Fanos et al. and Frick et al.^10−12^ Notably, the reduced levels of creatinine and taurine in infected newborns may suggest an overall disturbance in osmolyte balance, consistent with clinical outcomes of cCMV, such as nephropathy and developmental delays. This aligns with in vitro findings by Vastag et al.,^28^ showing intracellular taurine depletion in response to HCMV and HSV-1 infections due to virus-induced cell volume changes. However, Fanos et al.^12^ reported elevated taurine levels in their cohort. The discrepancy may stem from differences in study populations, as limited clinical data in Fanos et al.’s work makes direct comparison challenging. Finally, we observed an increase in ketone bodies, including 3-hydroxyisobutyrate, 4-hydroxybutyrate, and acetoacetate in the infected group. It is well established that circulating ketone body levels are slightly higher in healthy newborns compared to older children and adults, particularly during fasting. This phenomenon is likely due to metabolic adaptations in early life, including reliance on fat oxidation for energy and the high fat content of both human and formula milk. However, ketonuria is not typically observed in healthy newborns.^30,31^ Importantly, our cohort consisted of newborns who were not fasting at the time of sampling, as they were either breastfed or formula-fed.^32^ While we hypothesize that the observed increase in ketone bodies in HCMV-infected newborns may serve as a compensatory response to reduced ATP levels in infected cells, other metabolic responses to infection—such as increased energy demand or hepatic metabolic alterations—could have also contributed.

## Conclusions

This study demonstrates that urinary metabolomic profiling by ^1^H NMR spectroscopy combined with multivariate statistical analysis can effectively distinguish cCMV-infected newborns from healthy controls, highlighting its potential as a noninvasive diagnostic and prognostic tool. Although highly overlapping spectral profiles have been obtained, we were able to define distinct metabolic profiles between cCMV-infected newborns and healthy controls. The main limitation of this study is the relatively small sample size, and the single-center design may curtail the generalizability of the findings. As such, larger-scale prospective cohort studies are warranted to consolidate our findings. The data can also be integrated using a quantitative approach in a broader multicenter study. To mitigate these effects, we applied PLS-DA models coupled with a variable selection approach that combined VIP scores and Selectivity Ratio methods. Additionally, while the study identified important metabolic differences between infected and uninfected newborns, it remains unclear whether these differences are directly related to the viral infection or secondary to other factors, including age, type of delivery, gestational age, postnatal maturation, diet, and IUGR, which may have an impact on the neonatal urinary metabolome. Using our dataset, we could not obtain statistically significant models for any of these factors.

Thus, while our results are promising, this study should be considered a proof of concept, and larger multicenter studies with more diverse populations and longitudinal follow-up will be required to increase the statistical power and reliability of the model in predicting long-term outcomes in cCMV-infected newborns.

## Supporting Information Available

Table S1. Clinical and epidemiological data of the patients included in the study. Table S2. Clinical and epidemiological data of the controls included in the study. Table S3. List of metabolites identified in the ^1^H NMR spectra of patients’ and controls’ urine samples. Figure S1. Left. PCA scores plot of ^1^H NMR spectra obtained from cCMV infected patients (red squares, “P”) or healthy controls (green rhombs, “C”) relative to PC1. Right. Loadings plot relative to PC1. Figure S2. ROC Curve obtained from a PLS-DA model built considering all the samples and all the variables, with 2 latent variables. The AUROC value from cross-validation is 0.966. Figure S3. Representation of the variables selected using the VIP scores approach (green). All the acquired ^1^H NMR spectra are shown in various colors. Figure S4. ROC Curve obtained from a PLS-DA model built considering all the samples and only the variables selected using an approach that combines VIP scores and selectivity ratio methods. The model is built using 3 latent variables. The AUROC value obtained in cross-validation is 0.998 (PDF)
