# Supplementary material for: 1H NMR Urinary Metabolomics Profiling of Newborns with Congenital Human Cytomegalovirus Infection: Insights into Metabolic Alterations
Source: J Proteome Res. 2025 Mar 25;24(4):2112–20. doi: 10.1021/acs.jproteome.5c00017 (PMC11976849; doi:10.1021/acs.jproteome.5c00017)
Supplement: Supplementary file 1 — pr5c00017_si_001.pdf [file pr5c00017_si_001.pdf]

^{1}H-NMR urinary metabolomics profiling of newborns with congenital human cytomegalovirus infection: insights into metabolic alterations

Alessia Spadavecchia,[1‡] Marta Zoccarato,[2‡] Gaia Tedone,[1] Matteo Biolatti,[3] Valentina Dell'Oste,[3] Agata Leone,[1] Alessandro Cossard,[2] Mattia Sozzi,[4] Ilia Bresesti,[5] Enrico Bertino,[1] Roberto Gobetto,[2] Alessandra Coscia,[1] Angelo Gallo[2*]

[1]Neonatal Unit, Department of Public Health and Pediatric Sciences, University of Turin, Turin, 10126 Italy.

[2]Department of Chemistry, University of Turin, Turin, 10125 Italy.

[3]Department of Public Health and Pediatric Sciences, University of Turin, Turin, 10126 Italy.

[4]Department of Applied Science and Technology, Polytechnic of Turin, Turin, 10129 Italy.

[5]Division of Neonatology, "Filippo Del Ponte" Hospital, University of Insubria, Varese, 21100 Italy.

[‡]Alessia Spadavecchia and Marta Zoccarato contributed equally.

*To whom correspondence should be addressed: angelo.gallo@unito.it

SUPPLEMENTARY MATERIALS

Table S1. Clinical and epidemiological data of the patients included in the study.

**MOTHER**			
**Previous history of childbirth**		**Infection discovery**	
First Born	11 (30%)	*Unsuspected during pregnancy*	16 (44%)
Not firstborn	25 (70%)	*Serology*	17 (47%)
		Ultrasound abnormalities (not IUGR[a])	1 (3%)
		IUGR	0 (0%)
		Influenza syndrome	1 (3%)
		Acquired after birth	1 (3%)
**Type of infection**		**Trimester of infection**	
First	23 (65%)	*First*	12 (33%)
Relapse	5 (13%)	*Second*	7 (19%)
Postnatal	1 (3%)	*Third*	6 (16%)
N/A	7 (19%)	*Postnatal*	1 (3%)
		N/A	10 (29%)
**Antiviral therapy during pregnancy**			
Yes	4 (11%)		
No	31 (86%)		
N/A	1 (3%)		
**FETUS**			
**Fetal anomalies**		**Viral load on amniotic fluid**	
Yes	6 (17%)	*Positive*	2 (6%)
No	30 (83%)	*Negative*	4 (11%)
		N/A	30 (83%)
**CHILDBIRTH**			
**Preterm birth**		**Modality**	
Yes	4 (11%)	*Eutocic*	29 (80%)
No	32 (89%)	*Cesarean*	7 (20%)
**Reanimation at birth**			
Yes	2 (6%)		
No	33 (91%)		
N/A	1 (3%)		
**NEWBORN**			
**Sex**		**Birth weight**	
Male	16 (44%)	*> 10th pc*	27 (75%)
Female	20 (56%)	*3-10th pc*	3 (9%)
		< 3rd pc	6 (16%)
**Head circumference**		**Clinical symptoms at birth**	
> 10th pc	25 (69%)	*Yes*	6 (17%)
< 10t^{h} pc	11 (31%)	*No*	30 (83%)

INSTRUMENTAL EXAM ANOMALIES			
**Hearing**		**Cerebral Ultrasound**	
Normal	23 (63%)	*Normal*	16 (44%)
Pathologic	13 (37%)	*Pathologic*	20 (56%)
**Cerebral MRI**			
Normal	14 (38%)		
Pathologic	22 (62%)		

LABORATORY ANALYSES			
Platelets		***Neutrophils***	
Normal	27 (75%)	*Normal*	27 (75%)
Pathologic	4 (11%)	*Pathologic*	3 (9%)
N/A	5 (14%)	*N/A*	6 (16%)
**Hepatic functionality**			
Normal	27 (75%)		
Pathologic	1 (3%)		
N/A	8 (22%)		
**ANTIVIRAL THERAPY**			
Yes	16 (44%)		
No	20 (56%)		

Table S2. Clinical and epidemiological data of the controls included in the study.

MOTHER			
**Previous history of childbirth**		**Remote pathological anamnesis**	
Firstborn	10 (59%)	*Normal*	10 (59%)
Not firstborn	7 (41%)	*Abnormal*	7 (41%)
**Abnormalities in remote pathological anamnesis**		**Pregnancy anamnesis**	
Developmental dysplasia of the hip during infancy	2 (12%)	*Normal*	17 (100%)
Reynaud phenomenon and venous insufficiency	1 (6%)	*Abnormal*	0 (0%)
Beta-thalassemic trait	1 (6%)		
MTHFR mutation	2 (12%)		
Favism	1 (6%)		
**Therapies during pregnancy**		**HCMV infection status**	
Yes (Cardioaspirin until delivery)	1 (6%)	*Receptive*	16 (94%)
No	16 (94%)	*Immune*	1 (6%)
**FETUS**			
**Fetal anomalies**		**Ultrasound anomalies**	
Yes	0 (0%)	*Yes*	0 (0%)
No	17 (100%)	*No*	17 (100%)
**CHILDBIRTH**			
**Preterm birth**		**Modality**	

Yes	0 (0%)	*Eutocic*	14 (82%)
No	17 (100%)	*Cesarean*	7 (18%)
**Reanimation at birth**			
Yes	0 (0%)		
No	17 (100%)		
**NEWBORN**			
**Sex**		**Birth weight**	
Male	12 (70%)	*> 10th pc*	17 (100%)
Female	5 (30%)	*3-10th pc*	0 (0%)
		< 3rd pc	0 (0%)
**Head circumference**		**Clinical problems at birth**	
> 10th pc	15 (88%)	*Yes*	0 (0%)
< 10th pc	0 (0%)	*No*	17 (100%)
N/A	2 (12%)		
**Therapies during admission**		**Laboratory analysis during admission**	
Yes	0 (0%)	*Yes*	1 (6%)
No	17 (100%)	*G6PDH functionality*	1 (6%)
		No	16 (94%)
**Nutrition during admission**			
Breastfeeding	16 (94%)		
Standard infant formula	1 (6%)		
Donor human milk	0 (0%)		

Table S3. List of metabolites identified in the ^{1}H-NMR spectra of patients' and controls' urine samples.

Metabolites	*ppm*
1-methyl-nicotinamide	4.46 (s), 8.20 (t), 8.90 (d), 8.97 (d), 9.28 (t)
2-oxoglutarate	2.45 (t), 3.02 (t)
3-aminoisobutyrate	1.20 (d), 2.62 (m), 3.02 (m), 3.10 (m)
3-hydroxybutyrate	1.20 (d), 2.30 (m), 2.40 (m), 4.15 (m)
3-hydroxyisobutyrate	1.06 (d), 2.48 (m), 3.53 (m), 3.69 (m)
4-aminohippurate	3.90 (d), 6.90 (d), 7.80 (d), 8.20 (s)
4-hydroxyphenylacetate	3.44 (s), 6.85 (d), 7.16 (d)
4-hydroxybutyrate	1.74 (m), 2.21 (t), 3.57 (t)
Acetate	1.93 (s)
Acetoacetate	2.29 (s), 3.43 (s)
Acetone	2.24 (s)
Adipate	1.5 (m), 2.17 (t)
Alanine	1.5 (d), 3.8 (m)
Betaine	3.27 (s), 3.90 (s)
Caprilate	0.85 (d), 1.27 (m), 1.53 (m), 2.16 (t)
Cis-aconitate	3.12 (s), 5.77 (s)
Citrate	2.55 (dd), 2.70 (dd)

Creatine	3.02 (s), 3.91 (s)
Creatinine	3.02 (s), 4.06 (s)
Creatine phosphate	3.04 (s), 3.94 (s)
Dimethylamine	2.73 (s)
Ethanolamine	3.16 (t), 3.84 (t)
Ethanol	1.21 (t), 3.70 (q)
Formate	8.46 (s)
Fructose	3.57 (m), 3.70 (m), 3.82 (m), 3.90 (m), 4.01 (m), 4.10 (d)
Fumarate	6.53 (s)
Glycine	3.57 (s)
Glycolate	3.94 (s)
Glucose	3.23 (m), 3.40 (m), 3.48 (m), 3.74 (m), 3.83 (m), 3.90 (m), 4.26 (d), 5.28 (d)
Ibuprofen	0.86 (d), 1.41 (d), 1.82 (m), 2.46 (d), 3.60 (q), 7.22 (d), 7.27 (d)
Hippurate	4.02 (d), 7.55 (t), 7.65 (t), 7.85 (d), 8.50 (s)
Histamine	3.01 (t), 3.29 (t), 7.10 (s), 7.81 (s)
Lactate	1.32 (d), 4.10 (q)
Malonate	3.11 (s)
Mannitol	3.71 (m), 3.82 (m), 3.90 (dd)
Methanol	3.35 (s)
Myo-inositol	3.30 (t), 3.55 (t), 3.64 (dd), 4.08 (t)
N,N-dimethylglycine	2.93 (s), 3.71 (s)
Methylguanidine	2.83 (s), 6.98 (m)
N-phenyl-acetylglycine	3.67 (s), 7.36 (m), 7.44 (t), 7.99 (s)
Oxypurinol	8.19 (s)
Propyleneglycol	1.14 (m), 3.52 (m), 3.63 (q), 3.90 (d)
Sebacate	1.30 (s), 1.54 (m), 2.18 (t)
Suberate	1.30 (m), 1.55 (m), 2.18 (t)
Succinate	2.41 (s)
Taurine	3.25 (t), 3.42 (t)
Trans-aconitate	3.44 (s), 6.57 (s)
Threonine	1.32 (d), 3.58 (d), 4.25 (m)
Trimethylamine	2.87 (s)
Trimethylamine N-oxide	3.27 (s)
Urea	5.80 (s)
Valerate	0.87 (t), 1.29 (m), 1.51 (m), 2.17 (t)
Vanillate	3.90 (s), 6.93 (d), 7.44 (m), 7.52 (d)
Xanthine	7.96 (s)
Xanthosine	3.79 (m), 3.89 (m), 4.24 (m), 4.44 (m), 4.70 (m), 5.80 (d), 7.88 (s)

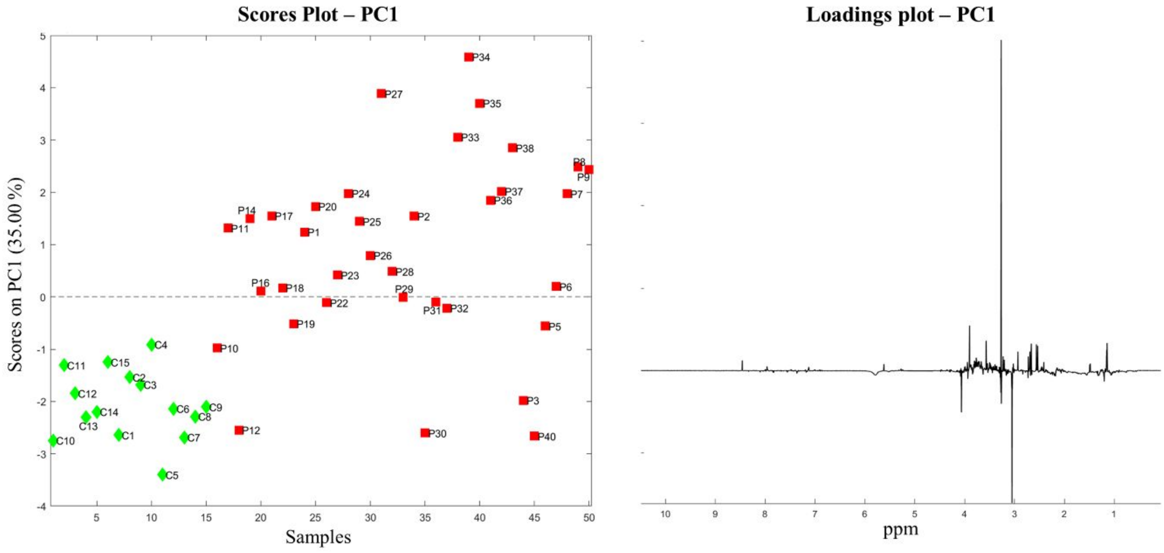

Figure S1. *Left.* PCA scores plot of ^{1}H-NMR spectra obtained from cCMV infected patients (red squares, "P") or healthy controls (green rhombs, "C") relative to PC1. *Right.* Loadings plot relative to PC1.

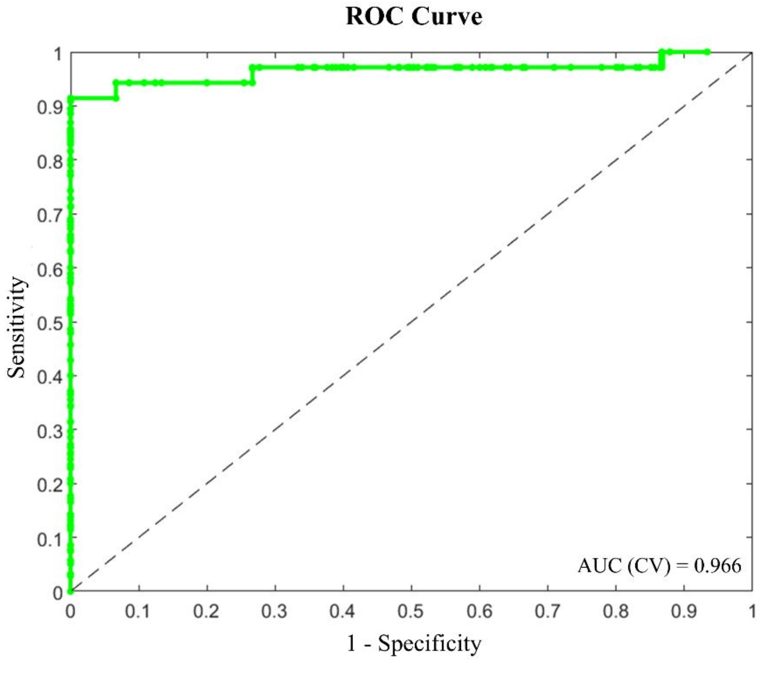

Figure S2. ROC Curve obtained from a PLS-DA model built considering all the samples and all the variables, with 2 latent variables. The AUROC value from cross-validation is 0.966.

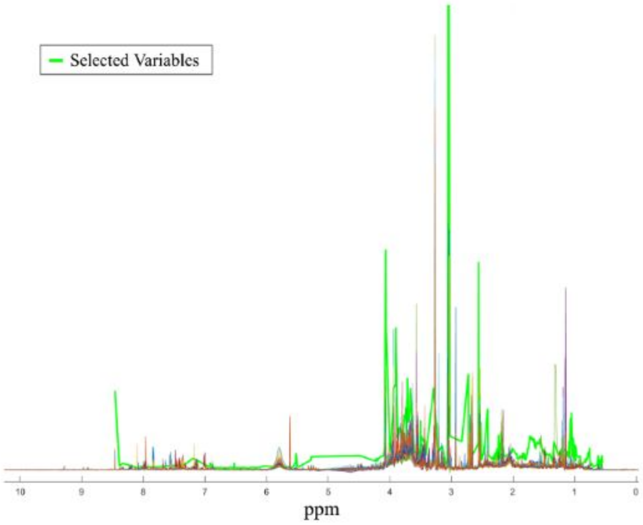

Figure S3. Representation of the variables selected using the VIP scores approach (green). All the acquired ^{1}H-NMR spectra are shown in various colours.

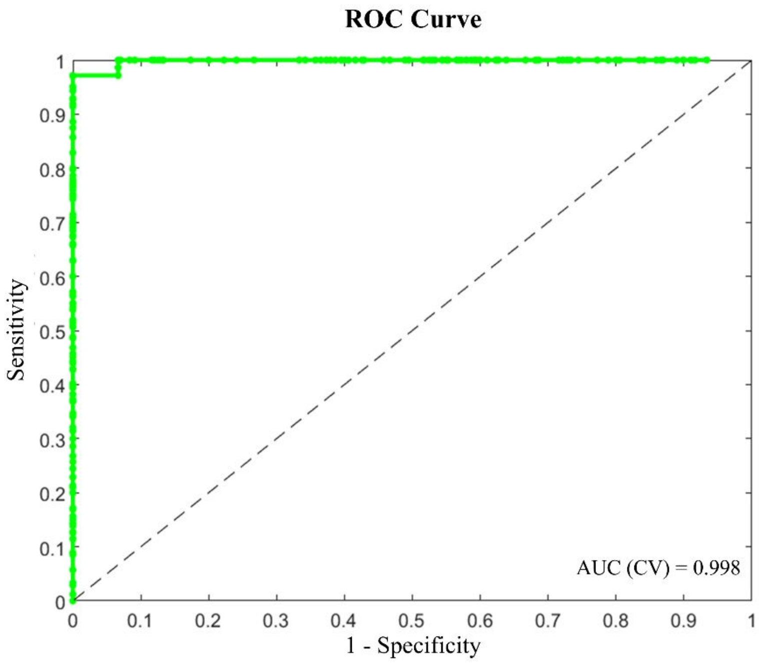

Figure S4. ROC Curve obtained from a PLS-DA model built considering all the samples and only the variables selected using an approach that combines VIP Scores and Selectivity Ratio methods. The model is built using 3 latent variables. The AUROC value obtained in cross-validation is 0.998.
